# Functional interaction of nicotinic acetylcholine receptors and Na^+^/K^+^ ATPase from *Locusta migratoria manilensis* (Meyen)

**DOI:** 10.1038/srep08849

**Published:** 2015-03-06

**Authors:** Haibo Bao, Huahua Sun, Youxin Xiao, Yixi Zhang, Xin Wang, Xiaoyong Xu, Zewen Liu, Jichao Fang, Zhong Li

**Affiliations:** 1 Key Laboratory of Integrated Management of Crop Diseases and Pests (Ministry of Education), College of Plant Protection, Nanjing Agricultural University, Weigang 1, Nanjing 210095, China; 2 Shanghai Key Laboratory of Chemical Biology, School of Pharmacy, East China University of Science and Technology, Meilong Road 130, Shanghai 200237, China; 3 Institute of Plant Protection, Jiangsu Academy of Agricultural Sciences, St. Zhongling 50, Nanjing 210014, China

## Abstract

Associated proteins are important for the correct functioning of nicotinic acetylcholine receptors (nAChRs). In the present study, a neonicotinoid-agarose affinity column was used to isolate related proteins from a solubilized membrane preparation from the nervous system of *Locusta migratoria manilensis* (Meyen). 1530 peptides were identified and most of them were involved in the membranous structure, molecular interaction and cellular communication. Among these peptides, Na^+^/K^+^ ATPase had the highest MASCOT score and were involved in the molecular interaction, which suggested that Na^+^/K^+^ ATPase and nAChRs might have strong and stable interactions in insect central nervous system. In the present study, functional interactions between nAChRs and Na^+^/K^+^ ATPase were examined by heterologous expression in *Xenopus* oocytes. The results showed that the activated nAChRs increased pump currents of Na^+^/K^+^ ATPase, which did not require current flow through open nAChRs. In turn, Na^+^/K^+^ ATPase significantly increased agonist sensitivities of nAChRs in a pump activity-independent manner and reduced the maximum current (*I_max_*) of nAChRs. These findings provide novel insights concerning the functional interactions between insect nAChRs and Na^+^/K^+^ ATPase.

Nicotinic acetylcholine receptors (nAChRs) are neurotransmitter receptors in the insect central nervous system and play an important role in insect physiology^1^,^2^. Insect nAChRs are also important targets for many kinds of insecticides, including neonicotinoids^3^, Spinosyns^4^, nereistoxin analogues^5^ and nicotine^6^. It is hoped that studies on the subunit composition and pharmacological properties of insect nAChRs will help in the development of novel insecticides and in their rational use and resistance management.

A widely used approach to studying the subunit composition and pharmacological properties of nAChRs is by constructing recombinant receptors in heterologous expression systems, such as *Xenopus* oocytes. Although there are plenty of reports on reconstructing nAChRs with insect subunits *in vitro*, frequently recombinant insect nAChRs have been successfully expressed only when insect α subunits are co-expressed with vertebrate β subunits^7^,^8^,^9^. It was believed that the unsuccessful heterologous expression of insect nAChRs was due to a requirement for one or several nAChR subunits which have not been identified. However, the completion of genome sequencing of some insect species^10^,^11^,^12^ suggested that the subunits or subunit combinations were not the key factors for such failure, because there are only α and β subunits in insects. In recent years, another hypothesis has been suggested that efficient expression of insect nAChRs required one or more associated proteins to assist in insects. Ly-6/neurotoxin (Lynx) and resistance to inhibitors of cholinesterase (Ric)-3 appear to be important chaperones for nAChRs, either in mammalian^13^ or in insects^14^,^15^. Lansdell et al.^16^ demonstrated the functional expression of only *Drosophila* nAChR subunits by the co-expression with Ric-3 in *Xenopus* oocytes, although the functional expression was inconsistent and failed in combinations. These studies indicated that there may be more associated proteins to assist nAChR subunit compositions in insects, including not only small soluble proteins but also membrane proteins. For example, some studies in mammals demonstrated that Na^+^/K^+^ ATPase (E.C. 3.6.1.37) was not only a plasma membrane ion pump contributing to the resting membrane potential^17^, but also a signal transducer^18^ and an interaction protein with nAChRs which affected the membrane electrogenesis^19^.

In the present study, a neonicotinoid-agarose affinity column was used to isolate proteins from a solubilized *L.* *migratoria manilensis* membrane preparation. The functions of the putative proteins were predicted by the gene ontology (GO) analysis. Na^+^/K^+^ ATPase, ranking first (with the highest MASCOT score) among the identified proteins, was then co-expressed with nAChRs in *Xenopus* oocytes to study the functional interaction between nAChRs and Na^+^/K^+^ ATPase.

## Results

### Protein isolation and identification

**Figure 1:**
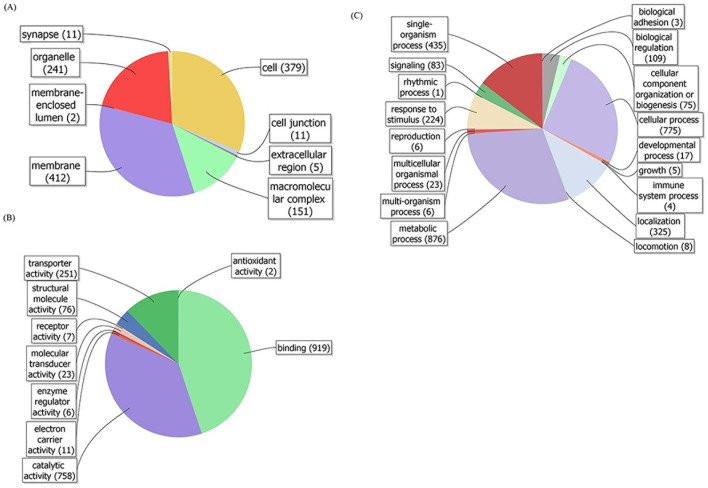
GO classification of identified proteins after Mascot search. Pie chart presentation of GO annotation was generated automatically by the web tool Blast2GO (http://www.blast2go.com/b2ghome) using the newest GO archive provided. The identified proteins were classified at the second level under three root GO domains: (A) cellular component, (B) molecular function and (C) biological process. For some cases, one identified proteins could be annotated into more than one GO term.

The membrane proteins were prepared from the nervous system, including brain, ventral nerve cord and ganglion subpharyngeale, of *L. migratoria manilensis* and a neonicotinoid-agarose affinity column was used to isolate related proteins from the solubilized membrane preparation. 1530 peptides were identified via a database search through the tandem mass spectrometry techniques, with MASCOT minimal peptides score of 37. Among these peptides, nAChR subunits were found (Table 1), which indicated that the neonicotinoid-agarose affinity column could isolate nAChRs from the solubilized membrane preparation. As reported previously^20^, if other proteins had strong and stable interaction with nAChRs caught by the column, these proteins would be also isolated from the solubilized membrane preparation when flowing through the column. Their cellular component association, molecular function and biological processes were analyzed using the gene ontology (GO) and 1311 annotated peptides were obtained (Figure 1). The two most enriched categories are membrane (containing 412 peptides) and cell (379) in GO domain of cellular component (Figure 1A), binding (919) and catalytic activity (758) in GO domain of molecular function (Figure 1B), and metabolic process (876) and cellular process (775) in GO domain of biology process (Figure 1C). Among these identified proteins, the Na^+^/K^+^ ATPase had the highest score and the most number of unique peptides (Table 1), which suggested that it might have strong and stable interaction with insect nAChRs.

### Clone and sequences analysis of Na^+^/K^+^ ATPase subunits

**Figure 2:**
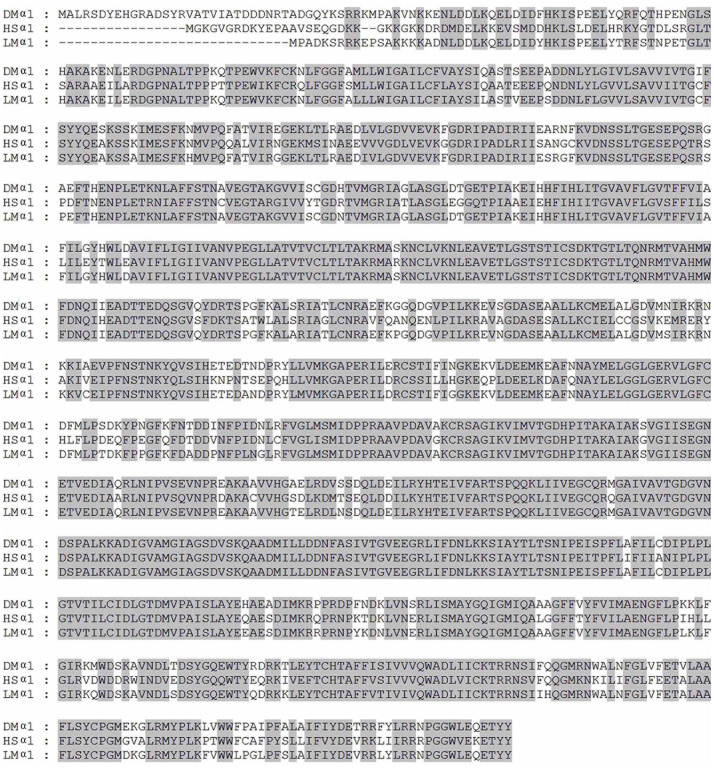
Alignment of amino acid sequences of Na^+^/K^+^ ATPase α1 subunit of *Drosophila melanogaster* (DM, Genebank accession NO. AAF55825), *Homo sapiens* (HS, P05023) and *Locusta migratoria* (LM, AHH35009). The sequences covered by shadow are identical. The “hinge” sequence is underlined. ATP phosphorylation site is indicated by triangles.

**Figure 3:**
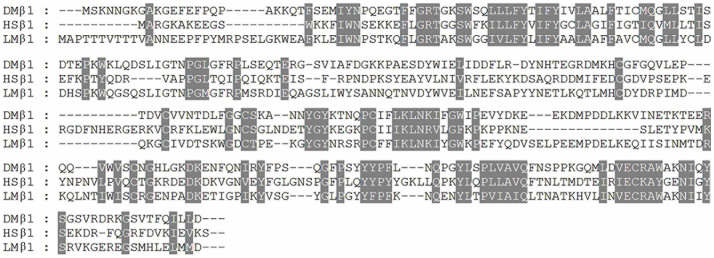
Alignment of amino acid sequences of Na^+^/K^+^ ATPase β1 subunit of *D*. *melanogaster* (DM, NP_477167), *H*. *sapiens* (HS, NP_001668) and *L*. *migratoria* *manilensis* (LM, AHH35012). The sequences covered by shadow are identical. Cysteine residues composed of S-S bridges are indicated by triangles.

For Na^+^/K^+^ ATPase, the functional enzyme unit is composed of at least one α subunit and one β subunit^21^,^22^. In order to construct a functional unit of *L. migratoria manilensis* Na^+^/K^+^ ATPase, subunits α1 and β1 were originally cloned in our laboratory for the subsequent studies. The nucleotide full length of subunit α1 is 3571 base pairs with an open reading frame (ORF) of 3039 base pairs, while subunit β1 is 1651 base pairs with an ORF of 978 base pairs. Analysis of the deduced amino acid sequence of subunit α1 revealed that it contained conserved “hinge” sequence and ATP phosphorylation sites that are signature motifs of P-type ATPase^23^,^24^ and had high similarities to α1 subunits of *Drosophila melanogaster* (91.8%) and *Homo sapiens* (84.8%) (Figure 2). Likewise, subunit β1 shared the rich cysteine residues composed of S-S bridges in extracellular domain which are signature motifs of β subunit of Na^+^/K^+^ ATPase^22^, though it only showed medium similarities to β1 subunits of *Drosophila melanogaster* (53.5%) and *Homo sapiens*(38.5%) (Figure 3).

### Effects of nAChRs on the heterologously co-expressed Na^+^/K^+^ ATPase

**Figure 4:**
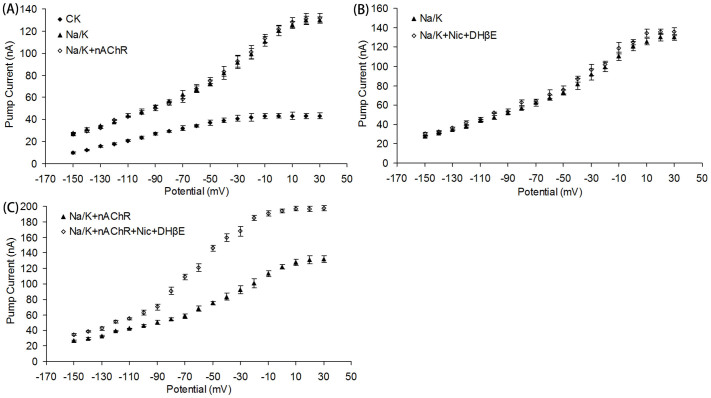
Effect of co-expressed nAChRs on pump currents of Na^+^/K^+^ ATPase. (A) Endogenous pump current of *Xenopus* oocytes as control (CK, solid squares), oocytes expressing Na^+^/K^+^ ATPase (solid triangles) and oocytes co-expressing Na^+^/K^+^ ATPase and nAChRs (open squares). (B) Pump currents of oocytes expressing Na^+^/K^+^ ATPase before (solid triangles) and after (open squares) the activation by 1 μM nicotine and 1 μM DHβE. (C) Pump currents of oocytes co-expressing Na^+^/K^+^ ATPase and nAChRs before (solid triangles) and after (open squares) the activation by 1 μM nicotine and 1 μM DHβE.

To investigate effects of nAChRs on the activity of Na^+^/K^+^ ATPase, the nAChRs (*L. migratoria manilensis* α1 and *R. norvegicus* β2) were co-expressed with Na^+^/K^+^ ATPase (α1 and β1) in *Xenopus* oocytes, and pump-mediated steady-state current was determined by the two-electrode voltage-clamp recording. The results showed that the co-expressed nAChRs had no effects on the activity of Na^+^/K^+^ ATPase directly (Figure 4A). However, when the nAChR agonist nicotine was added, the steady state current of Na^+^/K^+^ ATPase increased significantly (Figure 4C). DHβE, a specific inhibitor of nAChRs, was added to specifically and completely suppress the currents generated by nicotine on nAChRs^25^. Furthermore, we confirmed that nicotine and DHβE had no effects on Na^+^/K^+^ ATPase steady-state current directly (Figure 4B). Collectively, it was reasonable to conclude that the activated nAChRs could significantly increase the steady-state current of Na^+^/K^+^ ATPase when co-expressed in oocytes.

### Modulation of nAChRs sensitivity to agonist by Na^+^/K^+^ ATPase

**Figure 5:**
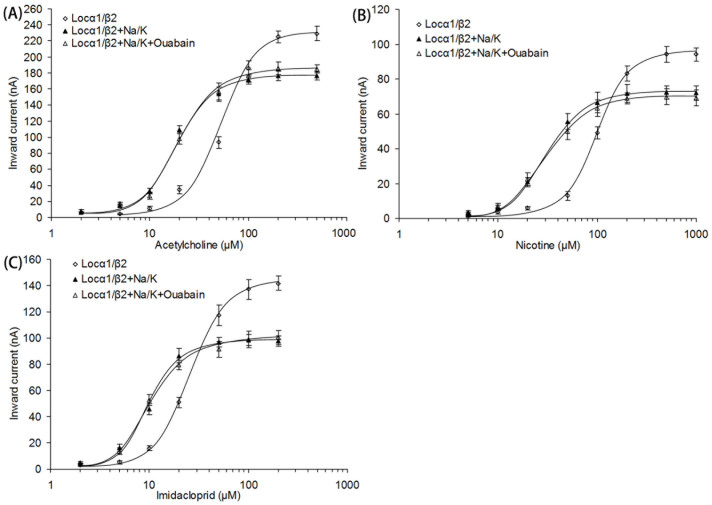
Effects of Na^+^/K^+^ ATPase on agonist sensitivities of nAChRs. (A) Does-response curves for acetylcholine. (B) Does-response curves for nicotine. (C) Does-response curves for imidacloprid. The curves from the oocytes expressing nAChRs (*L. migratoria manilensis* subunit α1 and *R. norvegicus* subunit β2) were indicated by open squares. The curves from the oocytes co-expressing nAChRs and Na^+^/K^+^ ATPase (subunit α1 and β1) were indicated by solid triangles (without ouabain) and open triangles (with ouabain).

To characterize the influence of Na^+^/K^+^ ATPase on nAChR function, nAChRs were expressed with or without Na^+^/K^+^ ATPase in *Xenopus* oocytes. The dose-response curves for acetylcholine (ACh) (Figure 5A) showed that Na^+^/K^+^ ATPase could increase ACh sensitivity, with *EC*_50_ of 63.21 ± 5.14 μM on oocytes only expressing nAChRs and 17.36 ± 2.49 μM on oocytes co-expressing nAChRs and Na^+^/K^+^ ATPase. The addition of ouabain (1 mM), a specific inhibitor of Na^+^/K^+^ ATPase to completely suppress the ion pump activity^26^, did not significantly change ACh sensitivity (*EC*_50_ = 21.50 ± 3.03 μM) on oocytes co-expressing nAChRs and Na^+^/K^+^ ATPase. The results of dose-response studies for nicotine and imidacloprid were similar to that of acetylcholine (Figure 5B and Figure 5C), which showed that co-expression of Na^+^/K^+^ ATPase could increase agonist sensitivities and such increases could not be removed by the addition of ouabain. In all dose-response studies, *I*_max_ values of agonists on oocytes only expressing nAChRs were much higher than that on oocytes co-expressing nAChRs and Na^+^/K^+^ ATPase.

## Discussion

Insect nAChRs have high affinity to neonicotinoids and we utilized this property to isolate nAChRs and related proteins possessing strong and stable interactions with nAChRs by the affinity chromatography. The protein extracts from insect nervous tissue membrane were applied to a neonicotinoid-affinity column to isolate proteins binding specificly to the neonicotinoid insecticide. If some other proteins have strong and stable interaction with nAChRs caught by the column, these proteins will be also isolated from the solubilized membrane preparation when flowing through the column, as reported previously^20^. The neonicotinoid-affinity column method had some advantages over traditional protein interaction screens, such as pulldown and yeast two-hybrid, to isolate interacted proteins of nAChRs, because the isolation process already indicated their native interactions^27^,^28^. The isolated protein complexes were analyzed by LC-MS/MS and more than 200 different proteins and protein complexes were identified. The GO analysis showed that most of the proteins participated in the membranous structure, molecular interaction and cellular communication. However, nonspecific binding of proteins to the column and also to neonicotinoid-column complex could not be completely ruled out. These nonspecific proteins could be mostly ruled out by the agarose affinity column without neonicotinoid insecticide and combination with other affinity column, such as α-bungarotoxin (α-BGT)-agarose affinity column^20^. Because of the possibility of nonspecific proteins, it is essential to confirm the interactions between nAChRs and other isolated proteins, with different methods, such as co-precipitation in native protein preparation or by studying functional interactions in a heterologous expression system. In the present study, functional interaction studies were performed to confirm the possible interaction between insect nAChRs and Na^+^/K^+^ ATPase.

Among these identified proteins, the existence of nAChR subunits indicated that the isolation was successful and agreed with the expectation^20^. In other identified proteins, Na^+^/K^+^ ATPase were found with the highest Mascot score in database searches. A feature of mass spectrometry is that proteins of high abundance and high molecular weight are much easier to identify. That is the probable reason why there was only one entry corresponding to a nAChR subunit and more than twenty (23 entries) corresponding to Na^+^/K^+^ ATPase. All Na^+^/K^+^ ATPase entries corresponded to α subunits, but none to β subunit, which might be also because of the much smaller molecular weight of Na^+^/K^+^ ATPase β subunit compared to α subunits. It is well known that the functional units of nAChRs and Na^+^/K^+^ ATPase are protein complexes. Thus, it is undeniable that the existence of other subunits involved in the interaction between nAChRs and Na^+^/K^+^ ATPase, even if they were not detected here.

If the nonspecific proteins binding to the column were presumed to be completely ruled out, the affinity chromatography method used in the present study identified a putative protein-protein interaction between nAChRs and Na^+^/K^+^ ATPase in the insect central nervous system. This possibility agrees with the previous studies on rat skeletal muscle, which showed that nAChRs could be co-immunoprecipitated with Na^+^/K^+^ ATPase, and Na^+^/K^+^ ATPase subunits could be co-immunoprecipitated with nAChRs^19^. The protein isolation results in the present study showed that nAChRs and Na^+^/K^+^ ATPase might have strong and stable interactions in insect central nervous system, because Na^+^/K^+^ ATPase had the highest Mascot score among all identified proteins. Because of the possibility of identifying nonspecific proteins, as mentioned above, a functional interaction study was performed to confirm the interaction between insect nAChRs and Na^+^/K^+^ ATPase. The activated nAChRs increased the pump current of Na^+^/K^+^ ATPase, which did not require currents through nAChRs. For this phenomenon, there were two possible explanations. 1) The stimulation of non-conducting and conformational changed nAChRs increased the expression and assembly of the Na^+^/K^+^ ATPase, similar with previous findings^29^. 2) The conformational change of nAChRs acted as a signal to enhance Na^+^/K^+^ ATPase activity. There is evidence that nAChRs are linked via adaptor proteins, such as linking the receptors to modulator, signaling enzymes, scaffolding proteins, kinases, and transcription factors^19^,^30^.

Most of the previous studies were focused on the regulation of nAChRs on the Na^+^/K^+^ ATPase^19^,^31^,^32^,^33^. In the present study, the effects of Na^+^/K^+^ ATPase on nAChRs function were also examined. The results showed that Na^+^/K^+^ ATPase significantly increased the sensitivity of nAChRs to agonists in a pump activity-independent manner. These results indicated that Na^+^/K^+^ ATPase was one of the associated proteins of insect nAChRs and could regulate nAChR function, such as agonist sensitivities. The results agreed with the previous studies in *Caenorhabditis elegans*, in which Na^+^/K^+^ ATPase could affect the clustering and localization of nAChRs independently from pump activity function^31^. Differently from the associated proteins identified in insects before, such as Lynx and Ric-3, Na^+^/K^+^ ATPase could change both agonist sensitivities (*EC*_50_ values) and maximum currents of nAChRs (*I*_max_). Lynx is an endogenous toxin-like modulator of nAChR^13^ and can increase the maximum currents of nAChRs to agonists, but had no significant effects on agonist sensitivities^14^. It has been shown that Lynx modulates nAChRs through the regulation of the efficiency of receptor folding and assembly. Likewise, Ric-3, a molecular chaperone of nAChRs, can enhance the function expression of nAChRs by folding and assembly of nAChR^34^,^35^. Although the interaction mechanisms between Na^+^/K^+^ ATPase and insect nAChRs was unclear, it was presumed that, like Lynx and Ric-3, Na^+^/K^+^ ATPase could increase membrane excitability of nAChRs to be more sensitive to exogenous and endogenous substances.

As multi-subunit transmembrane proteins, the assembly of nAChRs has been shown to be a slow and inefficient process^36^. To form functional native conformation, individual subunits must adopt an appropriate transmembrane topology and contribute to the appropriate subunit-subunit interactions^34^. It has been shown that nAChR folding, assembly and trafficking are assisted by several chaperone proteins, such as Lynx and Ric-3 (as mentioned above), 14-3-3 protein^37^,^38^,^39^, BiP^40^ and calnexin^41^,^42^. The present findings suggest that associated proteins could also exert considerable influences on nAChRs. The application of large-scale proteomics and high-throughput approaches have uncovered large proteins and protein complexes associated with nAChRs^43^,^44^. In mammals, a profusion of associated proteins of nAChRs have been identified with important roles in receptor folding, trafficking and assembly^44^. For insect nAChRs it is possible that co-expression of associate proteins help to oversome problems associated with inefficient heterologous expression. It is hoped that the present study will provide important information concerning the role of nAChR associated proteins in nAChRs function and and pharmacology in insects.

## Methods

### Insects

The *L. migratoria* *manilensis* were purchased from Hongguang insect breeding professional cooperative store, Nanjing (Jiangsu Province), China.

### Chemicals

Sequencing Grade Modified Trypsin was purchased from Promega Corporation (Madison, WI, USA). ECH Sepharose 4B was purchased from GE (Fairfield, CT, USA). Pierce® BCA Protein Assay Kit was purchased from Thermo Fisher Scientific Inc. (MA, USA). Bio-Scale MT Columns, acrylamide, Bis-Acrylamide, ReadyPrep™ 2-D Cleanup Kit, Silver Stain Plus Kit, Bio-Safe Coomassie Stain G250, Precision Plus Protein™ Dual Xtra Standards and Laemmli sample buffer were purchased from Bio-Rad Laboratories, Inc. (Hercules, CA, USA). mMESSAGE mMACHINE® T7 Transcription Kit was purchased from Life Technologies (Carlsbad, CA, USA). Clark Borosilicate Thin Wall with Filament GC150TF were purchased from Warner Instruments, Inc. (Hamden, CT, USA). Formic acid (FA), acetonitrile (ACN), acetylcholine (ACh), imidacloprid (Imi), dihydro-β-erythroidine (dHβE) and ouabain were purchased from Sigma-Aldrich, Inc. (St. Louis, MO, USA).

Nitenpyram was generously provided by Professor Zhong Li of East China University of Science and Technology.

### Synthesis of affinity precursor

**Figure 6:**
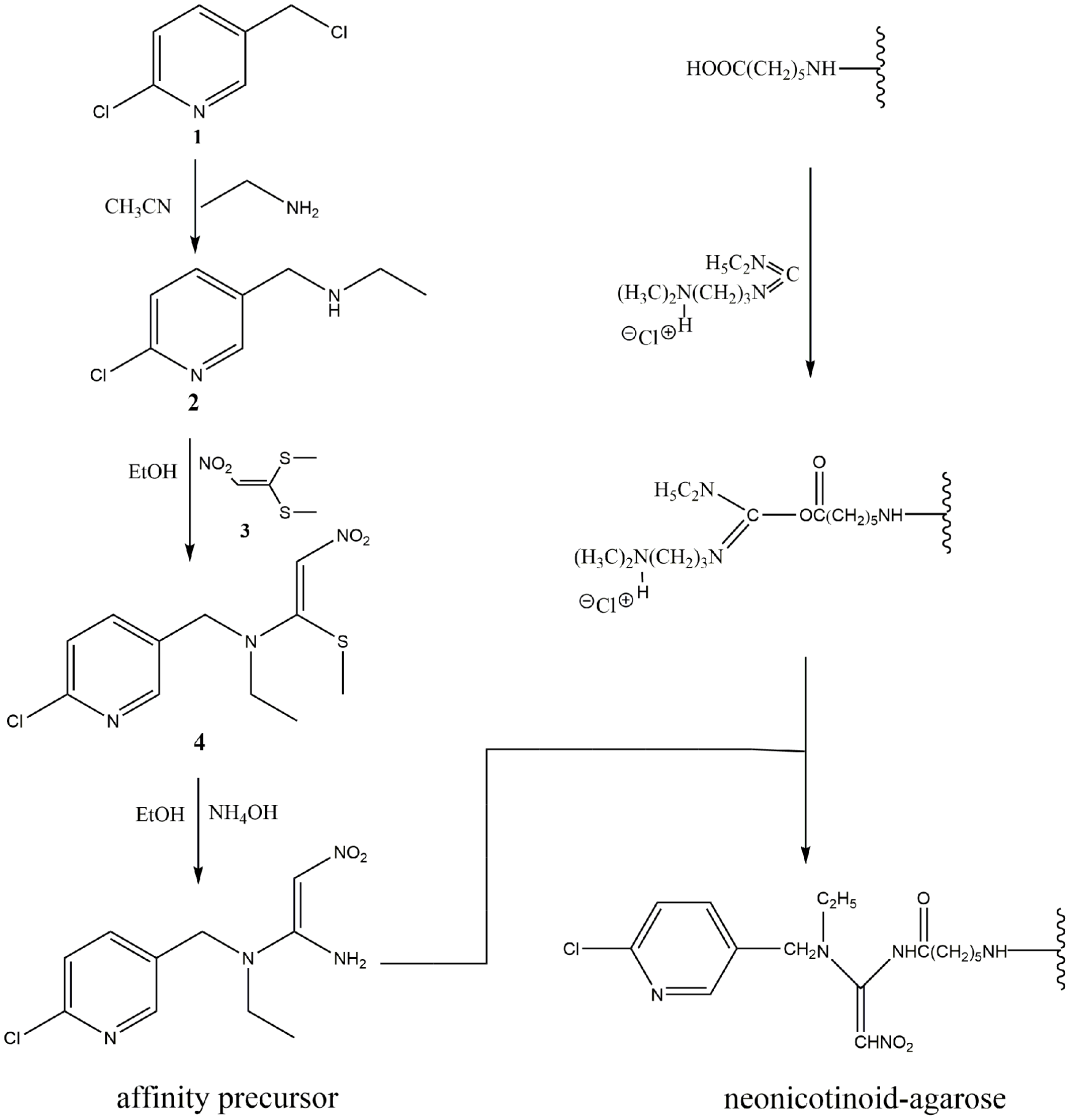
Synthesis of affinity precursor and preparation of neonicotinoid-agarose affinity gel.

Synthesis of these compounds (Figure 6) was based on the procedures of Motohiro Tomizawa et al.^20^ with modifications: 2-chloro-5-chloromethylpyridine (compound 1) was reacted with 10 equivalents of ethylamine (as a 65%–70% aqueous solution) in acetonitrile at ice temperature to give compound 2 in quantitative yield after extraction by dichloromethane. Compound 2 was then added dropwise in ethanol to a refluxing solution of 1 equivalents of 1, 1-bis (methylthio)-2-nitroethylene (compound 3) in ethanol, and the reaction was traced by thin layer chromatography. The product (compound 4) was isolated in 48.3% yield after concentration and purification by flash chromatography (silica gel) using petroleumether and ethylacetate for elution. Compound 4 in ethanol at reflux was treated with 10 molar equivalents of 58% ammonium hydroxide aqueous solution to obtain the affinity precursor in 52.1% yield after extraction by dichloromethane and vacuum filtration. Analytical data illustrated for the affinity precursor by the nuclear magnetic resonance signals in dimethyl sulfoxide-d_6_ (DMSO-d_6_) at 400 MHz for protons and 101 MHz for ^13^carbons are as follows: ^1^H NMR (400 MHz, DMSO-*d*_6_) δ 8.29 (d, *J* = 2.4 Hz, 1H), 7.68 (dd, *J* = 2.4 Hz, 8.4 Hz, 1H), 7.53 (d, *J* = 8.4 Hz, 1H), 6.57 (s, 1H), 4.65 (s, 2H), 3.41 (q, *J* = 6.8 Hz, 2H), 1.11 (t, *J* = 6.8 Hz, 3H). ^13^C NMR (101 MHz, DMSO-*d*_6_) δ = 157.2, 149.7, 148.8, 138.6, 132.5, 124.7, 98.7, 48.6, 44.5, 13.0. The elemental composition of the affinity precursor was confirmed by the exact mass measurement on high resolution electrospray ionization mass spectrometry: m/z C_10_H_13_N_4_O_2_^35^Cl calculated, 256.0727; found, 256.0729; C_10_H_13_N_4_O_2_^37^Cl calculated, 258.0698; found 258.0709.

Neonicotinoid-agarose affinity column (Figure 6) was prepared as description of Motohiro Tomizawa et al.^20^.

### Preparation of membrane of *L. migratoria manilensis*

The brain, ventral nerve cord and ganglion subpharyngeale were vivisected from adults of *L. migratoria* *manilensis* which were anaesthetized by ice. All above nervous tissues were collected into tubes, frozen with liquid nitrogen and stored at −80°C for later analysis. Subsequent steps were referred to Motohiro Tomizawa et al.^20^ with some modifications. The collected nervous tissues were homogenized at 10% (wt/vol) in 10 mM Na_2_HPO_4_-HCl buffer (pH 7.4) containing 100 mM NaCl, 5 mM EDTA, 3 mM EGTA, 0.1 mM 4-(2-Aminoethyl) benzenesulfonyl fluoride, and 0.02% sodium azide. The homogenate was centrifuged at 1,400 g for 15 min, and the resultant supernatant was centrifuged at 100,000 g for 60 min. The pellet was resuspended in the same type of buffer. TritonX-100 was then added at a final concentration of 1%, and the mixture was stirred at 4°C for 60 min. Unsolubihized material was removed by centrifugation at 100,000 g for 45 min. Protein concentration was determined by Pierce® BCA Protein Assay Kit.

### Affinity chromatography

The collection of eluates of *L. migratoria* *manilensis* nervous tissues followed the instructions of Motohiro Tomizawa et al.^20^ with some modifications. *L. migratoria* *manilensis* nervous tissues solubilized with 1% Triton X-l00 were incubated with the neonicotinoid gel in the column over night at 4°C. The amounts used were up to 5 mg of protein, at 1 mg protein/ml of gel. The column was then washed with 40 gel volumes of isolation buffer to remove nonspecifically bound proteins. The buffer in the column was drained while introducing the same volume of 50 μM nitenpyram in isolation buffer, and the column was incubated for 60 min at 4°C. Proteins selectively bound by the affinity gel were eluted with an additional 2 gel volumes of 50 μM nitenpyram. The eluates were concentrated with ReadyPrep™ 2-D Cleanup Kit and the purified protein precipitated was resuspended with an appropriate volume of Laemmli sample buffer for SDS-polyacrylamide gel electrophoresis (SDS-PAGE).

### SDS-PAGE

SDS-PAGE was performed using the small size 10% acrylamide gels (8.3 cm × 7.3 cm), 1 mm thick, which was casted with 30% Acrylamide/Bis solution (37.5:1). The protein samples (20 μl) were loaded into the gel well. As a standard, the Precision Plus Protein™ Dual Xtra Standards from Bio-Rad was used. Then, electrophoresis began with an initial voltage of 30 V and maintain at this voltage until the sample has completely entered the gel. Then the electrophoresis was carried out at a constant voltage of 200 V for 2 h. Gels were stained with Bio-Safe™ Coomassie G250 Stain. The sample lane of the gel was separated into two fractions at position of about 50 kD for liquid chromatography coupled with tandem mass spectrometry (LC-MS/MS) analysis.

### LC-MS/MS Analysis

LC-MS/MS analysis based on Orbitrap^45^. The procedures of LC-MS/MS analysis were referred to Su et al^46^ with some modifications. Each fraction was resuspended in buffer A (2% ACN, 0.1% FA) and centrifuged at 20,000 g for 15 min. 10 μl supernatant was loaded onto a 2 cm C18 trap column on a Dionex U-3000 Ultimate nano LC system by the auto sampler. Then, the peptides were eluted onto a self-packed resolving 10 cm analytical C18 column. The samples were loaded at 4 μl/min for 5 min and eluted with a 34 min gradient at 400 nl/min from 8% to 30% buffer B (98% ACN, 0.1% FA), then eluted by another 5 min gradient from 30% to 60%, followed by 3 min linear gradient to 80% B, and maintained at 80% B for 8 min, and finally returned to 5% B over 1 min. The peptides were subjected to nanoelectrospray ionization followed by tandem mass spectrometry (MS/MS) in a Q Exactive (Thermo scientific) coupled inline to the HPLC. Intact peptides were detected in the Orbitrap at a resolution of 60,000. Peptides were selected for MS/MS using the high-energy collision dissociation (HCD) operating mode with a normalized collision energy setting of 28%. Ion fragments were detected in the LTQ. A data-dependent procedure that alternated between one MS scan followed by ten MS/MS scans was applied for the ten most abundant precursor ions above a threshold ion count of 5,000 in the MS survey scan with the following Dynamic Exclusion settings: repeat counts, 2; repeat duration, 30 s; and exclusion duration, 120 s. The applied electrospray voltage was 1.8 kV. Automatic gain control (AGC) was used to prevent overfilling of the ion trap; 1 × 10^4^ ions were accumulated in the ion trap to generate HCD spectra. For MS scans, the m/z scan range was 350 to 2,000 Da.

### Database Search

The resulting LC-MS/MS spectra were searched against the NCBI insecta sequence databases with MASCOT software (Matrix Science, London, U.K.; version 2.3.01). For protein identification and quantification, a peptide mass tolerance of 15 ppm was allowed for intact peptide masses and 20 mmu for fragmented ions. One missed cleavage was allowed in the trypsin digests. The carbamidomethylation of cysteine was considered as a fixed modification, and the conversion of N-terminal glutamine to pyroglutamic acid and methionine oxidation were considered as variable modifications. All identified peptides had an ion score above the Mascot peptide identity threshold, and a protein was considered to be identified if at least one such unique peptide match was apparent for the protein. The significance threshold of protein identity is p < 0.05.

### Cloning of cDNAs

Through techniques of polymerase chain reaction (PCR) and rapid-amplification of cDNA ends (RACE), the full length cDNAs of the Na^+^/K^+^ ATPase subunits of *L. migratoria* *manilensis* were originally obtained by our laboratory and titled as α1 and β1 by phylogenetic homology. The information of the sequences has been released to the database of GeneBank. For the Na^+^/K^+^ ATPase subunits α1 and β1, the accession numbers are KF813096 and KF813099 respectively. The full length cDNA of nAChR subunit α1 of *L. migratoria* *manilensis* was also cloned base on the sequence (KF873578) from GeneBank.

### Expression and electrophysiological recording in *Xenopus* oocytes

The *L. migratoria manilensis* subunit α1 (KF813096) and subunit β1 (KF813099) of Na^+^/k^+^ ATPase, and subunit α1 (KF873578) of nAhCRs were subcloned into the expression vector pGH19 at sites of BamHIand XbaI, BamHI and ApaI, and EcoRI and XbaI, respectively. *Rattus norvegicus* β2 subunit (L31622) was also subcloned into the expression vector pGH19 as described previously^47^. The cRNAs of all subunits were generated as described previously^48^. *Xenopus* oocyte preparation and cRNA injection were performed as described previously^47^. Electrophysiological recordings were made using a two-electrode voltage clamp (Multiclamp 700B Amplifier; Axon Instruments, Foster, CA, USA) as previously described^47^. The measurement of the current of Na^+^/K^+^ ATPase was performed as described^49^.

### Statistical analysis

Differences in responses (currents), *I*_max_ and *EC*_50_ values were analyzed by one-way ANOVA with at least three repeats (different batches of oocytes from different frogs). Multiple comparisons between the groups were performed using S-N-K method. The level of significance for results was set at p < 0.05.

## Author Contributions

H.B., Z.W.L. and Y.Z. designed the experiments. H.B., H.S. and X.W. prepared samples and performed experiments. Y.X., X.X. and Z.L. helped with synthesis of neonicotinoid-agarose. H.B. performed the data analysis and prepared the manuscript. Z.W.L. and J.F. supervised the data analysis and edited the manuscript. All authors reviewed the manuscript.

## Figures and Tables

**Table 1:** Mascot search results of nAChR and Na^+^/K^+^ ATPase

*NCBI accession NO.*	*protein*	MASCOT score*	*Mass, kDa*	*NO. of Unique peptides*
gi|212513104	nAChR subunit alpha	65	17.8	2
gi|407731618	Na^+^/K^+^ ATPase alpha-subunit 1	15277	115.7	35
gi|407731570	Na^+^/K^+^ ATPase alpha-subunit 1	15150	116.7	33
gi|407731612	Na^+^/K^+^ ATPase alpha-subunit 1B, partial	15030	116.8	36
gi|407731614	Na^+^/K^+^ ATPase alpha-subunit 1A, partial	14861	112.6	35
gi|407731564	Na^+^/K^+^ ATPase alpha-subunit 1	14749	115.6	36
gi|407731588	Na^+^/K^+^ ATPase alpha-subunit 1	14663	116.4	35
gi|407731566	Na^+^/K^+^ ATPase alpha-subunit 1A, partial	14557	112.6	32
gi|399114499	Na^+^/K^+^ ATPase alpha subunit, partial	14364	88.9	29
gi|407731596	Na^+^/K^+^ ATPase alpha-subunit 1C, partial	14085	110.8	31
gi|332027641	Na^+^/K^+^ ATPase alpha-subunit	13855	123.1	33
gi|407731602	Na^+^/K^+^ ATPase alpha-subunit 1C, partial	13289	111.0	31
gi|407731600	Na^+^/K^+^ ATPase alpha-subunit 1	13250	113.3	33
gi|399114483	Na^+^/K^+^ ATPase alpha subunit, partial	12881	80.8	30
gi|407731578	Na^+^/K^+^ ATPase alpha-subunit 1	12450	112.4	29
gi|212512596	Na^+^/K^+^ ATPase alpha-subunit 1	12110	112.8	31
gi|373194435	Na^+^/K^+^ ATPase subunit alpha-3	11808	96.8	30
gi|212512149	Na^+^/K^+^ ATPase alpha-subunit 1	11668	115.7	29
gi|167862764	Na^+^/K^+^ ATPase subunit alpha	10767	81.1	22
gi|407731562	Na^+^/K^+^ ATPase alpha-subunit 1	10708	115.5	28
gi|307207574	Na^+^/K^+^ATPase alpha	10606	89.5	26
gi|407731616	Na^+^/K^+^ ATPase alpha-subunit 1	10450	115.5	27
gi|407731604	Na^+^/K^+^ ATPase alpha-subunit 1B, partial	10135	110.0	30
gi|407731574	Na^+^/K^+^ ATPase alpha-subunit 1	10134	112.5	23

*MASCOT minimal protein score = 37.

## References

[b1] Breer H., Kleene R. & Hinz G. Molecular-forms and subunit structure of the acetylcholine-receptor in the central-nervous-system of insects. J. Neurosci. 5, 3386–3392 (1985). 4078632 10.1523/JNEUROSCI.05-12-03386.1985 PMC6565241

[b2] Sattelle D. B. & Breer H. Cholinergic nerve-terminals in the central-nervous-system of insects - molecular aspects of structure, function and regulation. J. Neuroendocrinol. 2, 241–256 (1990). 19215342 10.1111/j.1365-2826.1990.tb00400.x

[b3] Matsuda K. *et al.* Neonicotinoids: Insecticides acting on insect nicotinic acetylcholine receptors. Trends Pharmacol. Sci. 22, 573–580 (2001). 11698101 10.1016/s0165-6147(00)01820-4

[b4] Geng C., Watson G. B. & Sparks T. C. Nicotinic Acetylcholine Receptors as Spinosyn Targets for Insect Pest Management *Target Receptors in* *The Control of Insect Pests*: *Part I*. Cohen E. (ed.) 101–210 (Academic Press, London, 2013).

[b5] Sattelle D. B. *et al.* Nereistoxin - actions on a CNS acetylcholine-receptor ion channel in the cockroach *Periplaneta americana*. J. Exp. Biol. 118, 37–52 (1985).

[b6] Harp P., Olivier K. & Pope C. Nicotine and organophosphate insecticides: Potential for interactive toxicity. FASEB J. 11, A209–A209 (1997).

[b7] Liu Z. *et al.* Heteromeric co-assembly of two insect nicotinic acetylcholine receptor alpha subunits: Influence on sensitivity to neonicotinoid insecticides. J. Neurochem. 108, 498–506 (2009). 19046356 10.1111/j.1471-4159.2008.05790.x

[b8] Bertrand D. *et al.* Physiological-properties of neuronal nicotinic receptors reconstituted from the vertebrate beta-2 subunit and *Drosophila* alpha-subunits. Eur. J. Neurosci. 6, 869–875 (1994). 8075828 10.1111/j.1460-9568.1994.tb00997.x

[b9] Liu Z. W. *et al.* A nicotinic acetylcholine receptor mutation conferring target-site resistance to imidacloprid in *Nilaparvata lugens* (brown planthopper). Proc. Natl. Acad. Sci. U. S. A. 102, 8420–8425 (2005). 15937112 10.1073/pnas.0502901102 PMC1150837

[b10] Adams M. D. *et al.* The genome sequence of *Drosophila melanogaster*. Science 287, 2185–2195 (2000). 10731132 10.1126/science.287.5461.2185

[b11] Nene V. *et al.* Genome sequence of *Aedes aegypti*, a major arbovirus vector. Science 316, 1718–1723 (2007). 17510324 10.1126/science.1138878 PMC2868357

[b12] Richards S. *et al.* The genome of the model beetle and pest *Tribolium castaneum*. Nature 452, 949–955 (2008). 18362917 10.1038/nature06784

[b13] Miwa J. M. *et al.* Lynx1, an endogenous toxin-like modulator of nicotinic acetylcholine receptors in the mammalian CNS. Neuron 23, 105–114 (1999). 10402197 10.1016/s0896-6273(00)80757-6

[b14] Liu Z., Cao G., Li J., Bao H. & Zhang Y. Identification of two lynx proteins in *Nilaparvata lugens* and the modulation on insect nicotinic acetylcholine receptors. J. Neurochem. 110, 1707–1714 (2009). 19619141 10.1111/j.1471-4159.2009.06274.x

[b15] Yang B. *et al.* Selectivity of lynx proteins on insect nicotinic acetylcholine receptors in the brown planthopper, *Nilaparvata lugens*. Insect Mol. Biol. 19, 283–289 (2009). 20002807 10.1111/j.1365-2583.2009.00981.x

[b16] Lansdell S. J., Collins T., Goodchild J. & Millar N. S. The *Drosophila* nicotinic acetylcholine receptor subunits d alpha 5 and d alpha 7 form functional homomeric and heteromeric ion channels. BMC Neurosci. 13 (2012). 10.1186/1471-2202-13-73 PMC3444433 22727315

[b17] Skou J. C. & Esmann M. The na,k-atpase. J. Bioenerg. Biomembr. 24, 249–261 (1992). 1328174 10.1007/BF00768846

[b18] Xie Z. & Askari A. Na^+^/k^+^-ATPase as a signal transducer. Eur. J. Biochem. 269, 2434–2439 (2002). 12027880 10.1046/j.1432-1033.2002.02910.x

[b19] Heiny J. A. *et al.* The nicotinic acetylcholine receptor and the Na^+^,K^+^-ATPase alpha 2 isoform interact to regulate membrane electrogenesis in skeletal muscle. J. Biol. Chem. 285, 28614–28626 (2010). 20595385 10.1074/jbc.M110.150961 PMC2937887

[b20] Tomizawa M., Latli B. & Casida J. E. Novel neonicotinoid-agarose affinity column for *Drosophila* and musca nicotinic acetylcholine receptors. J. Neurochem. 67, 1669–1676 (1996). 8858952 10.1046/j.1471-4159.1996.67041669.x

[b21] DeTomaso A. W., Xie Z. J., Liu G. & Mercer R. W. Expression, targeting, and assembly of functional Na,K-ATPase polypeptides in baculovirus-infected insect cells. J. Biol. Chem. 268, 1470–1478 (1993). 8380413

[b22] Martin D. W. Structure-function relationships in the Na^+^,K^+^-pump. Semin. Nephrol. 25, 282–291 (2005). 16139683 10.1016/j.semnephrol.2005.03.003

[b23] Mobasheri A. *et al.* Na^+^,k^+^-atpase isozyme diversity; comparative biochemistry and physiological implications of novel functional interactions. Biosci. Rep. 20, 51–91 (2000). 10965965 10.1023/a:1005580332144

[b24] Scheiner-Bobis G. The sodium pump: Its molecular properties and mechanics of ion transport. Eur. J. Biochem. 269, 2424–2433 (2002). 12027879 10.1046/j.1432-1033.2002.02909.x

[b25] Shoaib M., Zubaran C. & Stolerman I. P. Antagonism of stimulus properties of nicotine by dihydro-beta-erythroidine (dh beta e) in rats. Psychopharmacology 149, 140–146 (2000). 10805608 10.1007/s002139900348

[b26] Blanco G. Na,K-ATPase subunit heterogeneity as a mechanism for tissue-specific ion regulation. Semin. Nephrol. 25, 292–303 (2005). 16139684 10.1016/j.semnephrol.2005.03.004

[b27] Cho S. Y., Park S. G., Lee D. H. & Park B. C. Protein-protein interaction networks: From interactions to networks. J. Biochem. Mol. Biol. 37, 45–52 (2004). 14761302 10.5483/bmbrep.2004.37.1.045

[b28] Kabbani N., Woll M. P., Levenson R., Lindstrom J. M. & Changeux J. P. Intracellular complexes of the 2 subunit of the nicotinic acetylcholine receptor in brain identified by proteomics. Proc. Natl. Acad. Sci. U. S. A. 104, 20570–20575 (2007). 18077321 10.1073/pnas.0710314104 PMC2154472

[b29] Kragenbrink R., Higham S. C., Sansom S. C. & Pressley T. A. Chronic stimulation of acetylcholine receptors: Differential effects on Na^+^,K^+^-ATPase isoforms in a myogenic cell line. Synapse 23, 219–223 (1996). 8807750 10.1002/(SICI)1098-2396(199607)23:3<219::AID-SYN11>3.0.CO;2-U

[b30] Hezel M., de Groat W. C. & Galbiati F. Caveolin-3 promotes nicotinic acetylcholine receptor clustering and regulates neuromuscular junction activity. Mol. Biol. Cell 21, 302–310 (2009). 19940021 10.1091/mbc.E09-05-0381 PMC2808226

[b31] Doi M. & Iwasaki K. Na^+^/k^+^ atpase regulates the expression and localization of acetylcholine receptors in a pump activity-independent manner. Mol. Cell. Neurosci. 38, 548–558 (2008). 18599311 10.1016/j.mcn.2008.05.003 PMC2569892

[b32] Krivoi I. I. *et al.* On the functional interaction between nicotinic acetylcholine receptor and Na^+^,K^+^-ATPase. Pflugers Arch.-Eur. J. Physiol. 452, 756–765 (2006). 16636868 10.1007/s00424-006-0081-6

[b33] Chibalin A. V. *et al.* Chronic nicotine modifies skeletal muscle Na^+^,K^+^-ATPase activity through its interaction with the nicotinic acetylcholine receptor and phospholemman. PLoS One 7, e33719 (2012). 22442718 10.1371/journal.pone.0033719 PMC3307752

[b34] Millar N. S. Ric-3: A nicotinic acetylcholine receptor chaperone. Br. J. Pharmacol. 153, S177–S183 (2008). 18246096 10.1038/sj.bjp.0707661 PMC2268041

[b35] Lansdell S. J. *et al.* Host-cell specific effects of the nicotinic acetylcholine receptor chaperone Ric-3 revealed by a comparison of human and *Drosophila* Ric-3 homologues. BMC Neurosci. 105, 1573–1581 (2008). 10.1111/j.1471-4159.2008.05235.x 18208544

[b36] Green W. N. & Millar N. S. Ion-channel assembly. Trends Neurosci. 18, 280–287 (1995). 7571003

[b37] Jeanclos E. M., Lin L. & Anand R. Dynamic interaction of 14-3-3 with nicotinic alpha 4 beta 2 acetylcholine receptors. Society for Neuroscience Abstracts. 26, Abstract No.-235.235 (2000).

[b38] Jeanclos E. M. *et al.* The chaperone protein 14-3-3 eta interacts with the nicotinic acetylcholine receptor alpha 4 subunit - evidence for a dynamic role in subunit stabilization. J. Biol. Chem. 276, 28281–28290 (2001). 11352901 10.1074/jbc.M011549200

[b39] Exley R. *et al.* Chaperone protein 14-3-3 and protein kinase a increase the relative abundance of low agonist sensitivity human alpha 4 beta 2 nicotinic acetylcholine receptors in *xenopus* oocytes. J. Neurochem. 98, 876–885 (2006). 16787419 10.1111/j.1471-4159.2006.03915.x

[b40] Blount P. & Merlie J. P. Bip associates with newly synthesized subunits of the mouse muscle nicotinic receptor. J. Cell Biol. 113, 1125–1132 (1991). 2040645 10.1083/jcb.113.5.1125 PMC2289008

[b41] Gelman M. S., Chang W. S., Thomas D. Y., Bergeron J. J. M. & Prives J. M. Role of the endoplasmic-reticulum chaperone calnexin in subunit folding and assembly of nicotinic acetylcholine-receptors. J. Biol. Chem. 270, 15085–15092 (1995). 7797492 10.1074/jbc.270.25.15085

[b42] Chang W., Gelman M. S. & Prives J. M. Calnexin-dependent enhancement of nicotinic acetylcholine receptor assembly and surface expression. J. Biol. Chem. 272, 28925–28932 (1997). 9360963 10.1074/jbc.272.46.28925

[b43] Kabbani N., Woll M. P., Levenson R., Lindstrom J. M. & Changeux J.-P. Intracellular complexes of the β2 subunit of the nicotinic acetylcholine receptor in brain identified by proteomics. Proc. Natl. Acad. Sci. U. S. A. 104, 20570–20575 (2007). 18077321 10.1073/pnas.0710314104 PMC2154472

[b44] Jones A. K., Buckingham S. D. & Sattelle D. B. Proteins interacting with nicotinic acetylcholine receptors: Expanding functional and therapeutic horizons. Trends Pharmacol. Sci. 31, 455–462 (2010). 20674046 10.1016/j.tips.2010.07.001

[b45] Hu Q. Z. *et al.* The orbitrap: A new mass spectrometer. J. Mass Spectrom. 40, 430–443 (2005). 15838939 10.1002/jms.856

[b46] Su L. *et al.* Identification of novel biomarkers for sepsis prognosis via urinary proteomic analysis using itraq labeling and 2D-LC-MS/MS. PLoS One 8, e54237 (2013). 23372690 10.1371/journal.pone.0054237 PMC3553154

[b47] Zhang Y. *et al.* Functional co-expression of two insect nicotinic receptor subunits (Nl alpha 3 and Nl alpha 8) reveals the effects of a resistance-associated mutation (Nl alpha 3(Y151S)) on neonicotinoid insecticides. J. Neurochem. 110, 1855–1862 (2009). 19627438 10.1111/j.1471-4159.2009.06280.x

[b48] Liu Z. *et al.* A nicotinic acetylcholine receptor mutation (Y151S) causes reduced agonist potency to a range of neonicotinoid insecticides. J. Neurochem. 99, 1273–1281 (2006). 16981889 10.1111/j.1471-4159.2006.04167.x

[b49] Deng H. *et al.* Interactions of Na^+^,K^+^-ATPase and co-expressed delta-opioid receptor. Neurosci. Res. 65, 222–227 (2009). 19619588 10.1016/j.neures.2009.07.003

